# (*E*)-1-Methyl-2-styrylpyridinium iodide

**DOI:** 10.1107/S1600536809027810

**Published:** 2009-07-18

**Authors:** Hoong-Kun Fun, Kullapa Chanawanno, Suchada Chantrapromma

**Affiliations:** a X-ray Crystallography Unit, School of Physics, Universiti Sains Malaysia, 11800 USM, Penang, Malaysia; b Crystal Materials Research Unit, Department of Chemistry, Faculty of Science, Prince of Songkla University, Hat-Yai, Songkhla 90112, Thailand

## Abstract

In the title compound, C_14_H_14_N^+^·I^−^, the cation exists in an *E* configuration with respect to the ethenyl bond and is slightly twisted, the inter­planar angle between the pyridinium and phenyl rings of the cation being 4.8 (2)°. In the crystal packing, the cations are stacked in an anti­parallel fashion along the *a* axis by a π–π inter­action involving both pyridinium and phenyl rings; the centroid–centroid distance is 3.542 (3) Å. Each iodide ion is sandwiched between two cations. The cations and iodide anions are linked together by weak C—H⋯I inter­actions, giving rise to ladder-like ribbons along the *a* axis.

## Related literature

For bond-length data, see: Allen *et al.* (1987 ▶). For background to non-linear optical materials research, see: Wenseleers *et al.* (1998 ▶). For related structures, see: Chanawanno *et al.* (2008 ▶); Chantrapromma *et al.* (2009*a* ▶,*b* ▶); Fun *et al.* (2009 ▶). For the stability of the temperature controller used in the data collection, see: Cosier & Glazer, (1986 ▶).
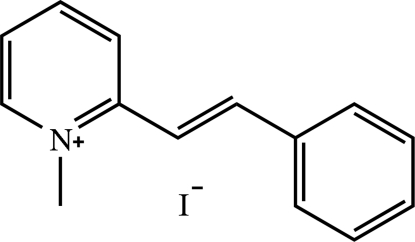


## Experimental

### 

#### Crystal data

C_14_H_14_N^+^·I^−^
*M* *_r_* = 323.16
Monoclinic, 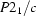

*a* = 7.0841 (1) Å
*b* = 10.0664 (2) Å
*c* = 19.1771 (3) Å
β = 109.017 (1)°
*V* = 1292.91 (4) Å^3^
*Z* = 4
Mo *K*α radiation
μ = 2.45 mm^−1^
*T* = 100 K
0.28 × 0.18 × 0.13 mm

#### Data collection

Bruker APEXII CCD area-detector diffractometer
Absorption correction: multi-scan (*SADABS*; Bruker, 2005 ▶) *T*_min_ = 0.552, *T*_max_ = 0.735
15764 measured reflections
3753 independent reflections
3186 reflections with *I* > 2σ(*I*)
*R*_int_ = 0.031

#### Refinement

*R*[*F* ^2^ > 2σ(*F* ^2^)] = 0.043
*wR*(*F* ^2^) = 0.107
*S* = 1.09
3753 reflections
146 parameters
H-atom parameters constrained
Δρ_max_ = 1.53 e Å^−3^
Δρ_min_ = −1.30 e Å^−3^

### 

Data collection: *APEX2* (Bruker, 2005 ▶); cell refinement: *SAINT* (Bruker, 2005 ▶); data reduction: *SAINT*; program(s) used to solve structure: *SHELXTL* (Sheldrick, 2008 ▶); program(s) used to refine structure: *SHELXTL*; molecular graphics: *SHELXTL*; software used to prepare material for publication: *SHELXTL* and *PLATON* (Spek, 2009 ▶).

## Supplementary Material

Crystal structure: contains datablocks global, I. DOI: 10.1107/S1600536809027810/wn2338sup1.cif

Structure factors: contains datablocks I. DOI: 10.1107/S1600536809027810/wn2338Isup2.hkl

Additional supplementary materials:  crystallographic information; 3D view; checkCIF report

## Figures and Tables

**Table 1:** Hydrogen-bond geometry (Å, °)

*D*—H⋯*A*	*D*—H	H⋯*A*	*D*⋯*A*	*D*—H⋯*A*
C1—H1*A*⋯I1^i^	0.93	3.05	3.799 (4)	139
C14—H14*A*⋯I1^ii^	0.96	3.04	3.996 (5)	173

Symmetry codes: (i) 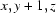; (ii) 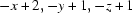.

**Fig. 1.**
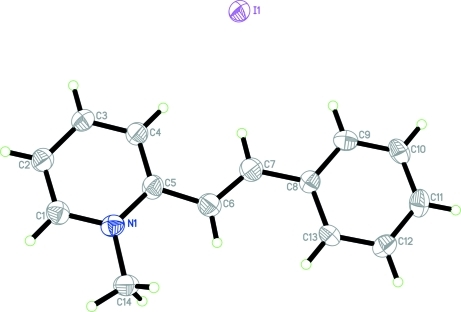
The molecular structure of the title compound, with 50% probability displacement ellipsoids and the atom-numbering scheme. Hydrogen atoms are drawn as spheres of arbitrary radius.

**Fig. 2.**
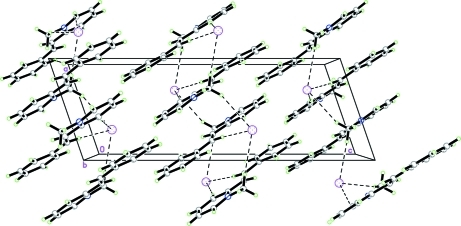
The crystal packing of the title compound, viewed down the b axis. Weak C—H···I interactions are shown as dashed lines.

## supplementary crystallographic information

### Comment

In the search for new materials capable of nonlinear optical (NLO) applications, many studies have focused on organic molecules containing highly polarizable π-conjugated backbones (Wenseleers *et al.*, 1998). During the course of our screening for NLO active organic compounds, we have previously reported the crystal structures of pyridinium derivatives (Chanawanno *et al.*, 2008; Chantrapromma *et al.*, 2009*a*,*b*); Fun *et al.*, 2009). In this paper we report the synthesis of the title compound whose crystal structure was undertaken in order to establish the conformation and crystal packing. The title compound crystallized in centrosymmetric space group *P*2_1_/*c* so it does not exhibit second-order nonlinear optical properties.

In the title compound, C_14_H_14_N^+^. I^-^ (Fig. 1), the cation exists in an *E* configuration with respect to the ethenyl C6═C7 double bond [1.347 (6) Å]; the torsion angle C5–C6–C7–C8 is 178.5 (4)°. The cation is slightly twisted, the interplanar angle between the pyridinium and phenyl rings being 4.8 (2)°. The bond distances in the cation have normal values (Allen *et al.*, 1987) and are comparable with closely related compounds (Chantrapromma *et al.*, 2009*a*,*b*; Fun *et al.*, 2009).

In the crystal packing (Fig. 2), the cations are stacked in an antiparallel fashion along the *a* axis by a π–π interaction with the Cg1···Cg2 distance = 3.542 (3) Å (symmetry code: 2-x, 1-y, 1-z); Cg1 and Cg2 are the centroids of the N1/C1–C5 and C8–C13 rings, respectively. Each iodide ion is sandwiched between two cations. The cations and iodide anions are linked together by weak C—H···I interactions, giving rise to ladder-like ribbons along the *a* axis (Table 1 and Fig. 2). The crystal structure is stabilized by C—H···I and π–π interactions.

### Experimental

The title compound was prepared by mixing 1:1:1 molar ratio solutions of 1,2-dimethylpyridinium iodide (2 g, 8.5 mmol), benzaldehyde (0.86 ml, 8.5 mmol) and piperidine (0.84 ml, 8.5 mmol) in methanol (40 ml). The resulting solution was refluxed for 5 hours under a nitrogen atmosphere. A pale yellow solid of the resulting compound was formed, this was then filtered and washed with diethyl ether. Yellow needle-shaped single crystals of the title compound suitable for *x*-ray structure determination were recrystallized from methanol by slow evaporation at room temperature after several weeks, Mp. 505-506 K. Details of the stability of the temperature controller used in the data collection have been published earlier (Cosier & Glazer, 1986).

### Refinement

All H atoms were positioned geometrically and allowed to ride on their parent atoms, with d(C-H) = 0.93 Å for aromatic C and CH and 0.96 Å for CH_3_ atoms. The *U*_iso_ values were constrained to be 1.5*U*_eq_ of the carrier atom for methyl H atoms and 1.2*U*_eq_ for the remaining H atoms. A rotating group model was used for the methyl groups. The highest residual electron density peak is located at 0.75 Å from I1 and the deepest hole is located at 0.67 Å from I1.

### Crystal data

**Table:**

C_14_H_14_N^+^·I^−^	*F*(000) = 632
*M**_r_* = 323.16	*D*_x_ = 1.660 Mg m^−^^3^
Monoclinic, *P*2_1_/*c*	Melting point = 505–506 K
Hall symbol: -P 2ybc	Mo *K*α radiation, λ = 0.71073 Å
*a* = 7.0841 (1) Å	Cell parameters from 3753 reflections
*b* = 10.0664 (2) Å	θ = 2.3–30.0°
*c* = 19.1771 (3) Å	µ = 2.45 mm^−^^1^
β = 109.017 (1)°	*T* = 100 K
*V* = 1292.91 (4) Å^3^	Block, pale yellow
*Z* = 4	0.28 × 0.18 × 0.13 mm

### Data collection

**Table:**

Bruker APEXII CCD area-detector diffractometer	3753 independent reflections
Radiation source: sealed tube	3186 reflections with *I* > 2σ(*I*)
graphite	*R*_int_ = 0.031
φ and ω scans	θ_max_ = 30.0°, θ_min_ = 2.3°
Absorption correction: multi-scan (*SADABS*; Bruker, 2005)	*h* = −8→9
*T*_min_ = 0.552, *T*_max_ = 0.735	*k* = −14→14
15764 measured reflections	*l* = −26→26

### Refinement

**Table:**

Refinement on *F*^2^	Primary atom site location: structure-invariant direct methods
Least-squares matrix: full	Secondary atom site location: difference Fourier map
*R*[*F*^2^ > 2σ(*F*^2^)] = 0.043	Hydrogen site location: inferred from neighbouring sites
*wR*(*F*^2^) = 0.107	H-atom parameters constrained
*S* = 1.09	*w* = 1/[σ^2^(*F*_o_^2^) + (0.037*P*)^2^ + 4.8574*P*] where *P* = (*F*_o_^2^ + 2*F*_c_^2^)/3
3753 reflections	(Δ/σ)_max_ < 0.001
146 parameters	Δρ_max_ = 1.53 e Å^−^^3^
0 restraints	Δρ_min_ = −1.30 e Å^−^^3^

### Special details

**Table:**

Experimental. The crystal was placed in the cold stream of an Oxford Cryosystems Cobra open-flow nitrogen cryostat (Cosier & Glazer, 1986) operating at 100.0 (1) K.
Geometry. All esds (except the esd in the dihedral angle between two l.s. planes) are estimated using the full covariance matrix. The cell esds are taken into account individually in the estimation of esds in distances, angles and torsion angles; correlations between esds in cell parameters are only used when they are defined by crystal symmetry. An approximate (isotropic) treatment of cell esds is used for estimating esds involving l.s. planes.
Refinement. Refinement of F^2^ against ALL reflections. The weighted R-factor wR and goodness of fit S are based on F^2^, conventional R-factors R are based on F, with F set to zero for negative F^2^. The threshold expression of F^2^ > 2sigma(F^2^) is used only for calculating R-factors(gt) etc. and is not relevant to the choice of reflections for refinement. R-factors based on F^2^ are statistically about twice as large as those based on F, and R- factors based on ALL data will be even larger.

### Fractional atomic coordinates and isotropic or equivalent isotropic displacement parameters (Å^2^)

**Table:**

	*x*	*y*	*z*	*U*_iso_*/*U*_eq_	
I1	0.70838 (4)	0.18319 (3)	0.382571 (14)	0.03436 (10)	
N1	0.6191 (5)	0.7789 (4)	0.45127 (18)	0.0298 (7)	
C1	0.5059 (6)	0.8350 (4)	0.3870 (2)	0.0327 (8)	
H1A	0.4830	0.9261	0.3851	0.039*	
C2	0.4245 (6)	0.7599 (5)	0.3248 (2)	0.0332 (8)	
H2A	0.3463	0.7993	0.2811	0.040*	
C3	0.4607 (6)	0.6242 (4)	0.3281 (2)	0.0305 (8)	
H3A	0.4094	0.5718	0.2863	0.037*	
C4	0.5727 (6)	0.5682 (4)	0.3936 (2)	0.0316 (8)	
H4A	0.5958	0.4772	0.3962	0.038*	
C5	0.6529 (7)	0.6466 (4)	0.4568 (2)	0.0328 (8)	
C6	0.7642 (7)	0.5934 (4)	0.5286 (2)	0.0346 (9)	
H6A	0.7946	0.6493	0.5694	0.041*	
C7	0.8250 (6)	0.4660 (5)	0.5384 (2)	0.0350 (9)	
H7A	0.7885	0.4131	0.4964	0.042*	
C8	0.9420 (6)	0.4001 (4)	0.6074 (2)	0.0272 (7)	
C9	0.9771 (6)	0.2630 (4)	0.6079 (2)	0.0299 (8)	
H9A	0.9310	0.2151	0.5641	0.036*	
C10	1.0797 (6)	0.1984 (4)	0.6727 (3)	0.0335 (9)	
H10A	1.1008	0.1073	0.6724	0.040*	
C11	1.1507 (7)	0.2682 (5)	0.7377 (2)	0.0365 (9)	
H11A	1.2225	0.2247	0.7810	0.044*	
C12	1.1146 (7)	0.4048 (5)	0.7385 (2)	0.0341 (9)	
H12A	1.1590	0.4516	0.7826	0.041*	
C13	1.0130 (6)	0.4704 (4)	0.6736 (2)	0.0291 (8)	
H13A	0.9920	0.5615	0.6742	0.035*	
C14	0.7074 (7)	0.8689 (5)	0.5147 (2)	0.0364 (9)	
H14A	0.8469	0.8492	0.5367	0.055*	
H14B	0.6409	0.8564	0.5505	0.055*	
H14C	0.6917	0.9593	0.4980	0.055*	

### Atomic displacement parameters (Å^2^)

**Table:**

	*U*^11^	*U*^22^	*U*^33^	*U*^12^	*U*^13^	*U*^23^
I1	0.04036 (17)	0.03000 (15)	0.02839 (14)	0.00091 (11)	0.00527 (11)	−0.00118 (10)
N1	0.0326 (17)	0.0315 (17)	0.0280 (15)	−0.0039 (14)	0.0137 (14)	−0.0016 (13)
C1	0.0287 (19)	0.034 (2)	0.036 (2)	0.0047 (16)	0.0115 (16)	0.0009 (16)
C2	0.0278 (19)	0.040 (2)	0.0309 (19)	0.0078 (17)	0.0080 (16)	0.0005 (17)
C3	0.0273 (18)	0.037 (2)	0.0278 (17)	−0.0012 (16)	0.0093 (15)	−0.0019 (16)
C4	0.035 (2)	0.028 (2)	0.0306 (18)	−0.0052 (16)	0.0094 (16)	0.0010 (15)
C5	0.038 (2)	0.0277 (19)	0.0304 (19)	−0.0067 (16)	0.0087 (17)	0.0050 (15)
C6	0.037 (2)	0.032 (2)	0.035 (2)	0.0006 (17)	0.0126 (17)	0.0000 (16)
C7	0.037 (2)	0.033 (2)	0.035 (2)	−0.0014 (17)	0.0115 (17)	0.0006 (17)
C8	0.0288 (18)	0.0266 (18)	0.0270 (16)	−0.0042 (15)	0.0100 (14)	0.0036 (14)
C9	0.0308 (19)	0.0301 (19)	0.0333 (19)	−0.0038 (16)	0.0166 (16)	−0.0037 (15)
C10	0.031 (2)	0.0222 (18)	0.047 (2)	0.0010 (15)	0.0130 (18)	0.0064 (16)
C11	0.033 (2)	0.036 (2)	0.036 (2)	−0.0011 (18)	0.0055 (17)	0.0119 (18)
C12	0.037 (2)	0.038 (2)	0.0268 (18)	−0.0043 (18)	0.0097 (16)	−0.0017 (16)
C13	0.0315 (19)	0.0222 (17)	0.0345 (19)	0.0014 (15)	0.0118 (16)	0.0002 (14)
C14	0.047 (2)	0.030 (2)	0.032 (2)	−0.0036 (19)	0.0130 (18)	−0.0053 (16)

### Geometric parameters (Å, °)

**Table:**

N1—C5	1.352 (5)	C7—H7A	0.9300
N1—C1	1.355 (5)	C8—C13	1.397 (5)
N1—C14	1.481 (5)	C8—C9	1.402 (6)
C1—C2	1.370 (6)	C9—C10	1.381 (6)
C1—H1A	0.9300	C9—H9A	0.9300
C2—C3	1.387 (6)	C10—C11	1.376 (6)
C2—H2A	0.9300	C10—H10A	0.9300
C3—C4	1.369 (6)	C11—C12	1.399 (7)
C3—H3A	0.9300	C11—H11A	0.9300
C4—C5	1.402 (6)	C12—C13	1.384 (6)
C4—H4A	0.9300	C12—H12A	0.9300
C5—C6	1.448 (6)	C13—H13A	0.9300
C6—C7	1.347 (6)	C14—H14A	0.9600
C6—H6A	0.9300	C14—H14B	0.9600
C7—C8	1.470 (6)	C14—H14C	0.9600
			
C5—N1—C1	121.3 (4)	C13—C8—C9	118.8 (4)
C5—N1—C14	121.4 (4)	C13—C8—C7	121.3 (4)
C1—N1—C14	117.3 (4)	C9—C8—C7	119.8 (4)
N1—C1—C2	121.2 (4)	C10—C9—C8	120.6 (4)
N1—C1—H1A	119.4	C10—C9—H9A	119.7
C2—C1—H1A	119.4	C8—C9—H9A	119.7
C1—C2—C3	119.0 (4)	C11—C10—C9	120.4 (4)
C1—C2—H2A	120.5	C11—C10—H10A	119.8
C3—C2—H2A	120.5	C9—C10—H10A	119.8
C4—C3—C2	119.4 (4)	C10—C11—C12	119.8 (4)
C4—C3—H3A	120.3	C10—C11—H11A	120.1
C2—C3—H3A	120.3	C12—C11—H11A	120.1
C3—C4—C5	120.8 (4)	C13—C12—C11	120.2 (4)
C3—C4—H4A	119.6	C13—C12—H12A	119.9
C5—C4—H4A	119.6	C11—C12—H12A	119.9
N1—C5—C4	118.4 (4)	C12—C13—C8	120.2 (4)
N1—C5—C6	117.8 (4)	C12—C13—H13A	119.9
C4—C5—C6	123.8 (4)	C8—C13—H13A	119.9
C7—C6—C5	122.3 (4)	N1—C14—H14A	109.5
C7—C6—H6A	118.8	N1—C14—H14B	109.5
C5—C6—H6A	118.8	H14A—C14—H14B	109.5
C6—C7—C8	128.1 (4)	N1—C14—H14C	109.5
C6—C7—H7A	116.0	H14A—C14—H14C	109.5
C8—C7—H7A	116.0	H14B—C14—H14C	109.5
			
C5—N1—C1—C2	−1.3 (6)	C4—C5—C6—C7	10.0 (7)
C14—N1—C1—C2	177.6 (4)	C5—C6—C7—C8	178.5 (4)
N1—C1—C2—C3	−0.4 (6)	C6—C7—C8—C13	−2.8 (7)
C1—C2—C3—C4	1.4 (6)	C6—C7—C8—C9	174.6 (4)
C2—C3—C4—C5	−0.8 (6)	C13—C8—C9—C10	0.2 (6)
C1—N1—C5—C4	1.9 (6)	C7—C8—C9—C10	−177.3 (4)
C14—N1—C5—C4	−176.9 (4)	C8—C9—C10—C11	−0.7 (6)
C1—N1—C5—C6	−175.9 (4)	C9—C10—C11—C12	1.5 (7)
C14—N1—C5—C6	5.3 (6)	C10—C11—C12—C13	−1.9 (7)
C3—C4—C5—N1	−0.8 (6)	C11—C12—C13—C8	1.5 (6)
C3—C4—C5—C6	176.8 (4)	C9—C8—C13—C12	−0.6 (6)
N1—C5—C6—C7	−172.4 (4)	C7—C8—C13—C12	176.9 (4)

### Hydrogen-bond geometry (Å, °)

**Table:**

*D*—H···*A*	*D*—H	H···*A*	*D*···*A*	*D*—H···*A*
C1—H1A···I1^i^	0.93	3.05	3.799 (4)	139
C14—H14A···I1^ii^	0.96	3.04	3.996 (5)	173

Symmetry codes: (i) *x*, *y*+1, *z*; (ii) −*x*+2, −*y*+1, −*z*+1.
